# Effects of a Novel Selective Peroxisome Proliferator-Activated Receptor α Modulator, Pemafibrate, on Metabolic Parameters: A Retrospective Longitudinal Study

**DOI:** 10.3390/biomedicines10020401

**Published:** 2022-02-08

**Authors:** Hidekatsu Yanai, Hisayuki Katsuyama, Mariko Hakoshima

**Affiliations:** Department of Diabetes, Endocrinology and Metabolism, National Center for Global Health and Medicine Kohnodai Hospital, Chiba 272-8516, Japan; d-katsuyama@hospk.ncgm.go.jp (H.K.); d-hakoshima@hospk.ncgm.go.jp (M.H.)

**Keywords:** liver function, non-alcoholic fatty liver disease, neurological diseases, pemafibrate, peroxisome proliferator-activated receptor-alpha

## Abstract

The modulation of peroxisome proliferator-activated receptors (PPARs), the superfamily of steroid–thyroid–retinoid nuclear receptors, is expected to induce an amazing crosstalk between energy-demanding organs. Here, we aimed to study the effects of the novel selective PPARα modulator, pemafibrate, on metabolic parameters in patients with dyslipidemia. We retrospectively studied patients who had taken pemafibrate and compared metabolic parameters at baseline with the data at 3, 6 and 12 months after the start of pemafibrate. Serum triglyceride significantly decreased and high-density lipoprotein-cholesterol significantly increased at 3, 6 and 12 months after the start of pemafibrate. Serum aspartate aminotransferase levels significantly decreased at 3 and 6 after the start of pemafibrate as compared with baseline. Serum alanine aminotransferase and gamma-glutamyl transferase significantly decreased and albumin significantly increased after 3, 6 and 12 months. HbA1c levels significantly decreased after 3 months. Further, serum uric acid significantly decreased after 12 months. Such metabolic favorable changes due to pemafibrate were significantly correlated with changes in serum lipids. In conclusion, we observed a significant improvement of liver function, HbA1c and serum uric acid along with an amelioration of dyslipidemia after the start of pemafibrate.

## 1. Introduction

The metabolic syndrome due to insulin resistance poses a global challenge as societies become increasingly urbanized, sedentary and obese. A cluster of risk factors for cardiovascular disease (CVD) have become known as the metabolic syndrome. The risk factors include raised blood pressure, atherogenic dyslipidemia such as raised triglyceride (TG) and lowered high-density lipoprotein cholesterol (HDL-C), raised fasting glucose, and central obesity [1]. Beyond lifestyle intervention, the modulation of the nuclear receptor peroxisome proliferator-activated receptor (PPAR) is a therapeutic option to improve atherogenic dyslipidemia and prevent CVD.

The modulation of peroxisome proliferator-activated receptors (PPARs) which belong to the superfamily of steroid–thyroid–retinoid nuclear receptors [2] may induce an amazing crosstalk between organs, because PPARs are transcription factors activated by specific ligands and play an important role during cell signaling. PPARs participate in the regulation of lipid metabolism, inflammation and the development of atherosclerosis or diabetes. Furthermore, therapeutic potential of PPARs agonists for neurological disease has been also suggested [3]. PPARs have three isoforms; PPARα, PPARγ and PPARβ/δ [4]. PPARα is abundant in energy-demanding tissues, such as liver, kidney, cardiac and skeletal muscles; PPARγ is predominantly found in adipose tissue, macrophages and large intestine, whereas PPARβ/δ is more ubiquitous in distribution [5,6]. 

Fibrates reduce serum TG by lowering hepatic apo CIII production and by increasing lipoprotein lipase (LPL)-mediated lipolysis and fatty acid (FA) oxidation, by mediating PPARα, and also elevate HDL-C levels via transcriptional induction of synthesis of HDL apolipoprotein AI and AII [7]. Clinical trials of fibrates have shown that serum TG decreased by about 25 to 50% and that serum HDL-C levels increased by 5 to 20% [8,9,10]. In addition, fibrates were reported to reduce the risk of CVD [10,11]. 

However, fibrates have been associated with increased risk of liver damage and increased levels of serum creatinine, the marker for renal dysfunction [12,13]. Because most fibrates are excreted through the kidney, excretion of fibrates is diminished in patients with renal dysfunction [10,13]. Therefore, fibrates are contraindicated in patients with renal dysfunction. 

Pemafibrate is a novel member of the selective PPARα modulator family that was designed to have a higher PPARα agonistic activity and selectivity than existing PPARα agonists (such as fibrates) [5,14]. In the previous study, pemafibrate was administered for 52 weeks to 189 patients with hypertriglyceridemia and an estimated glomerular filtration rate (eGFR) ≥ 45 mL/min/1.73 m^2^ on statin, or regardless of eGFR when statin was not administered [15]. There were no significant changes in eGFR over time in any stages of chronic kidney disease (CKD) [15]. Further, pemafibrate showed a good safety profile and efficacy in correcting lipid abnormalities in a broad range of patients, including those with CKD. Most fibrates are metabolized mainly by the kidneys, whereas pemafibrate is metabolized mainly by the liver [16].

Here, we aimed to study the influences of pemafibrate on metabolic parameters including the markers for liver dysfunction and eGFR in patients with dyslipidemia.

## 2. Materials and Methods

### 2.1. Study Population

The study protocol was approved by the Ethics Committee of the National Center for Global Health and Medicine (NCGM-S-004344-00), and the study was performed in accordance with the Declaration of Helsinki.

We retrospectively picked up patients who had taken pemafibrate from June 2018 to August 2021 and compared metabolic parameters at baseline with the data at 3, 6 and 12 months after the start of pemafibrate. Information about medical history and medication were obtained via an electronic medical record. Such information included age, gender, body weight, body mass index (BMI), systolic and diastolic blood pressures, comorbidities, and treatments for type 2 diabetes, hypertension, dyslipidemia and hyperuricemia. Informed consent was obtained by the opt-out approach. According to the diagnostic criteria of the Japan Diabetes Society, the Japanese Society of Hypertension and Japanese Society of Gout and Uric & Nucleic Acids, we defined type 2 diabetes as taking anti-diabetic drugs and/or HbA1c > 6.5%; hypertension as taking anti-hypertensive drugs and/or systolic blood pressure ≥ 140 mmHg and/or diastolic blood pressure ≥ 90 mmHg; and hyperuricemia as taking uric acid (UA) lowering drugs and/or serum UA ≥ 7.0 mg/dL, respectively.

### 2.2. Laboratory Measurements

Serum alanine aminotransferase (ALT), aspartate aminotransferase (AST), and gamma-glutamyl transferase (GGT) were measured by a modified Japan Society of Clinical Chemistry (JSCC) reference method. Serum albumin was measured by the modified bromocresol purple (BCP) method. Serum uric acid (UA) was measured by the uricase peroxidase method. Plasma glucose was measured by the hexokinase UV method. Hemoglobin A1c (HbA1c) was measured by automated enzyme-linked immunosorbent assays (TOSOH, Tokyo, Japan). Serum creatinine, TG, HDL-C and low-density lipoprotein-cholesterol (LDL-C) were determined enzymatically. Estimated glomerular filtration rate (eGFR) was calculated by using the Chronic Kidney Disease Epidemiology Collaboration (CKD–EPI) formula. 

### 2.3. Statistical Analysis

Statistical analyses were performed by using SPSS version 23 (IBM Co., Ltd., Chicago, IL, USA). All values are expressed as the mean ± standard deviation except for sex. The Wilcoxon signed-rank test was used to statistically analyze comparison in metabolic parameters between before and after the start of pemafibrate. Correlations between changes in two parameters were statistically analyzed by the Pearson’s correlation. *p* value of <0.05 was considered statistically significant.

## 3. Results

### 3.1. Baseline Characteristics of Patients Studied

We found 246 patients, and baseline characteristics for patients who had taken pemafibrate are shown in Table 1. Pemafibrate was prescribed to patients with hypertriglyceridemia (TG > 150 mg/dL). The mean BMI was over 25 kg/m^2^, indicating that our study included a relatively large number of overweight patients. Almost half of our patients had type 2 diabetes and hypertension, and one-third of patients were complicated with hyperuricemia. One-third to one-fourth of patients had taken dipeptidyl peptidase-4 inhibitors, and/or metformin and/or sodium-glucose co-transporter 2 inhibitors as the treatment for type 2 diabetes. One-third of patients had taken angiotensin receptor blockers and/or calcium antagonists as the treatments for hypertension. One-third of patients had taken stain in addition to pemafibrate as the treatments for dyslipidemia. Twenty patients had undergone the switching from fenofibrate to pemafibrate. One-fifth of patients were treated by UA lowering drugs. 

### 3.2. Correlations between Metabolic Parameters at Baseline

Body weight values are significantly and positively correlated with diastolic blood pressure (r = 0.182, *p* = 0.012), serum ALT (r = 0.274, *p* < 0.0001), UA (r = 0.372, *p* < 0.0001), HbA1c (r = 0.189, *p* = 0.009) and TG (r = 0.141, *p* = 0.046) levels. Body weight values are also significantly and negatively correlated with serum HDL-C levels (r = −0.15, *p* = 0.038). Serum TG levels are significantly and positively correlated with diastolic blood pressure (r = 0.2, *p* = 0.004), serum GGT (r = 0.148, *p* = 0.03), UA (r = 0.3, *p* < 0.001), HbA1c (r = 0.227, *p* = 0.001), and are significantly and negatively correlated with serum HDL-C levels (r = −0.32, *p* < 0.001). Serum non-HDL-C levels are significantly and positively correlated with diastolic blood pressure (r = 0.279, *p* < 0.001), serum ALT (r = 0.169, *p* = 0.022), UA (r = 0.277, *p* < 0.001), plasma glucose (r = 0.235, *p* = 0.001) and HbA1c (r = 0.312, *p* < 0.001) levels, and are significantly and negatively correlated with serum HDL-C levels (r = −0.285, *p* < 0.001).

### 3.3. Changes in Metabolic Parameters after the Start of Pemafibrate

#### 3.3.1. Changes in Metabolic Parameters after the Start of Pemafibrate in All Patients

These are shown in Table 2. 

Body weight and systolic and diastolic blood pressures did not show any changes. Serum TG and non-HDL-C levels significantly decreased and HDL-C level significantly increased at 3, 6 and 12 months after the start of pemafibrate as compared with baseline. Serum AST levels significantly decreased at 3 and 6 after the start of pemafibrate as compared with baseline. Serum ALT and GGT levels significantly decreased and albumin level significantly increased after 3, 6 and 12 months. Serum UA levels significantly decreased at 12 months after the start of pemafibrate as compared with baseline. Furthermore, HbA1c levels significantly decreased after 3 months. Plasma glucose and eGFR did not show any changes.

#### 3.3.2. Changes in Metabolic Parameters after the Start of Pemafibrate in Patients with and without the Treatment Using Sodium-Glucose Co-Transporter 2 Inhibitors (SGLT2i)

Because we previously reported that SGLT2i improves atherogenic dyslipidemia, liver function and serum UA in addition to glucose-lowering [17,18,19,20], we analyzed changes in metabolic parameters after the start of pemafibrate by dividing patients into patients who received SGLT2i and patients who did not receive SGLT2i (Table 3). 

We found no differences in changes in TG, HDL-C, non-HDL-C, ALT and GGT between the two groups. A significant increase in LDL-C after 3 and 12 months and a significant decrease in serum UA were observed in patients who did not receive SGLT2i. A significant decrease in AST after 3 and 12 months and a significant decrease in plasma glucose and HbA1c were observed in patients who received SGLT2i. Serum albumin significantly increased in patients who did not receive SGLT2i after 3, 6 and 12 months, but a significant increase in albumin was observed in patients who received SGLT2i only after 3 months. 

### 3.4. Correlations between Changes in Metabolic Parameters after the Start of Pemafibrate

#### 3.4.1. Correlations between Changes in Serum Lipids

The changes in serum TG were negatively correlated with changes in HDL-C (r = −0.311, *p* < 0.001; r = −0.311, *p* < 0.001; r = −0.315, *p* = 0.001 at 3, 6 and 12 months, respectively) and positively correlated with changes in non-HDL-C (r = 0.324, *p* < 0.001; r = 0.343, *p* < 0.001; r = 0.286, *p* = 0.005 at 3, 6 and 12 months, respectively) at 3, 6 and 12 months after the start of pemafibrate. 

#### 3.4.2. Correlations among Changes in the Markers for Liver Function

The changes in serum AST were positively correlated with changes in serum ALT (r = 0.718, *p* < 0.001; r = 0.753, *p* < 0.001; r = 0.752, *p* = 0.001 at 3, 6 and 12 months, respectively) and GGT (r = 0.471, *p* < 0.001; r = 0.339, *p* < 0.001; r = 0.433, *p* < 0.001 at 3, 6 and 12 months, respectively) at 3, 6 and 12 months after the start of pemafibrate. The changes in serum albumin were not correlated with other markers for liver dysfunction (AST, ALT and GGT) at 3 and 6 months after the start of pemafibrate. However, the changes in serum albumin were negatively correlated with changes in serum GGT (r = −0.239, *p* = 0.017) at 12 months after the start of pemafibrate.

#### 3.4.3. Correlations between Changes in Serum Lipids and Changes in the Markers for Liver Function

**Figure 1:**
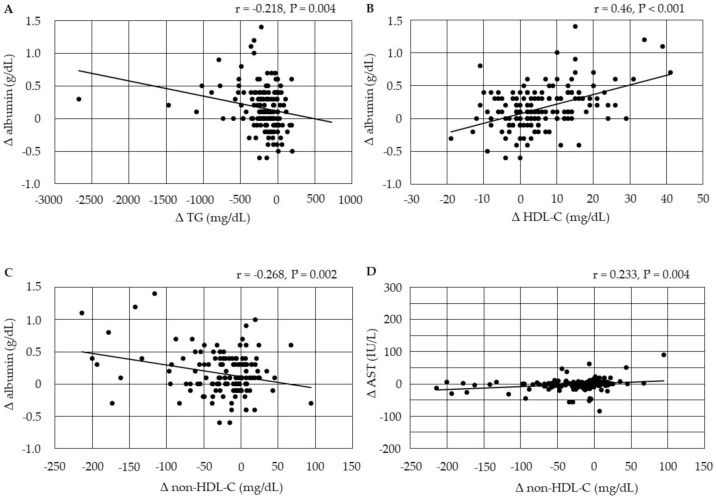
(**A**–**D**) Correlations between changes in serum lipids and changes in markers for liver function at 3 months after the start of pemafibrate. AST, aspartate aminotransferase; HDL-C, high-density lipoprotein-cholesterol; non-HDL-C, non-high-density lipoprotein-cholesterol; TG, triglyceride.

The changes in serum albumin were negatively correlated with changes in serum TG and non-HDL-C, and were positively correlated with changes in HDL-C at 3 months after the start of pemafibrate (Figure 1). Further, the changes in serum AST were positively correlated with changes in non-HDL-C after 3 months. 

**Figure 2:**
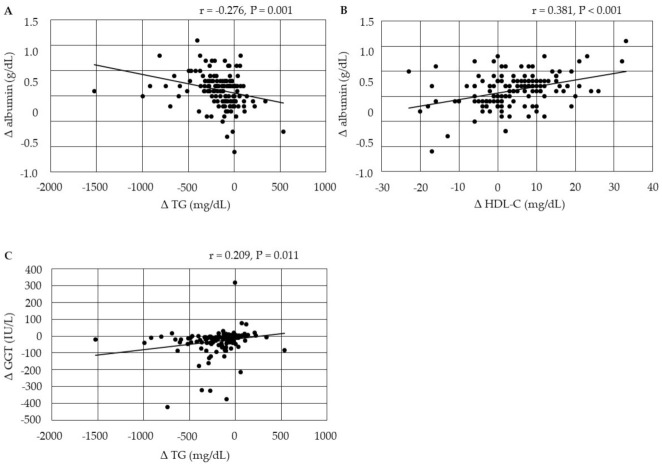
(**A**–**C**) Correlations between changes in serum lipids and changes in markers for liver function at 6 months after the start of pemafibrate. GGT, gamma-glutamyl transferase; HDL-C, high-density lipoprotein-cholesterol; non-HDL-C, non-high-density lipoprotein-cholesterol; TG, triglyceride.

The changes in serum albumin were negatively correlated with changes in serum TG, and were positively correlated with changes in HDL-C at 6 months after the start of pemafibrate (Figure 2). The changes in serum GGT were positively correlated with changes in serum TG after 6 months.

**Figure 3:**
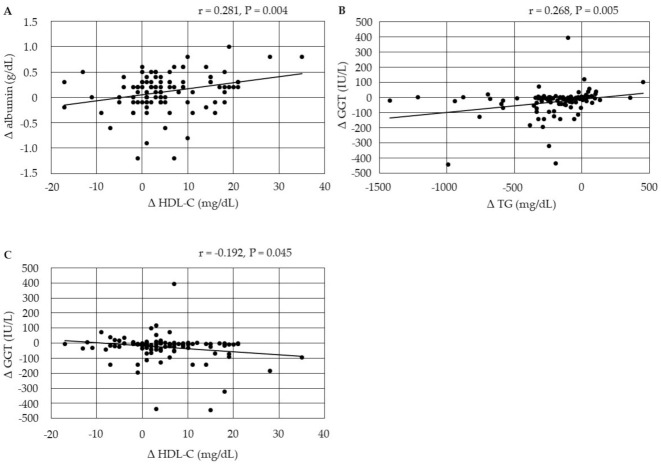
(**A**–**C**) Correlations between changes in serum lipids and changes in markers for liver function at 12 months after the start of pemafibrate. GGT, gamma-glutamyl transferase; HDL-C, high-density lipoprotein-cholesterol; TG, triglyceride.

The changes in serum albumin were positively correlated with changes in HDL-C at 12 months after the start of pemafibrate (Figure 3). The changes in serum GGT were positively correlated with changes in serum TG, and were negatively correlated with changes in HDL-C after 12 months.

#### 3.4.4. Correlations between Changes in Serum Lipids and Changes in HbA1c after the Start of Pemafibrate

**Figure 4:**
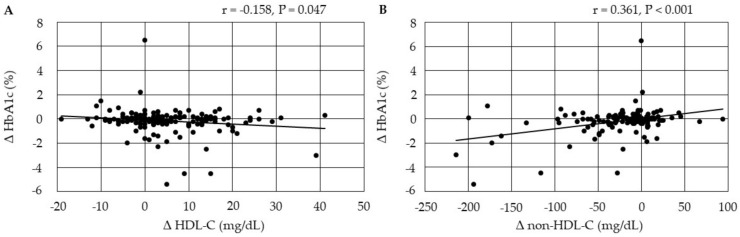
(**A**,**B**) Correlations between changes in serum lipids and changes in HbA1c at 3 months after the start of pemafibrate. HDL-C, high-density lipoprotein-cholesterol; non-HDL-C, non-high-density lipoprotein-cholesterol.

Changes in HbA1c were negatively correlated with changes in HDL-C, and were positively correlated with changes in non-HDL-C at 3 months after the start of pemafibrate (Figure 4).

#### 3.4.5. Correlations between Changes in Serum Lipids and Changes in Serum UA after the Start of Pemafibrate

**Figure 5:**
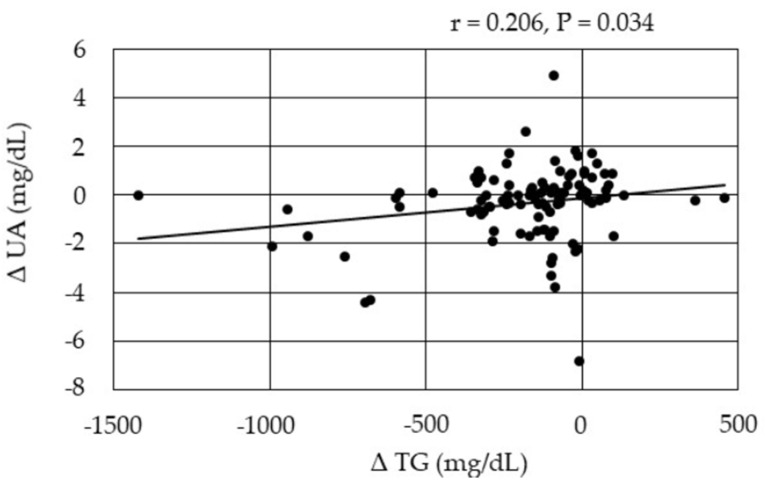
Correlations between changes in serum lipids and changes in UA at 12 months after the start of pemafibrate. TG, triglyceride; UA, uric acid.

Changes in serum UA were positively correlated with change in TG at 12 months after the start of pemafibrate (Figure 5).

## 4. Discussion

The increasing prevalence of the metabolic syndrome/insulin resistance puts a very large population at risk of developing non-alcoholic fatty liver disease (NAFLD) [21]. Our study included a relatively large number of patients with overweight and such patients showed atherogenic dyslipidemia which was commonly observed in insulin resistance. 

**Figure 6:**
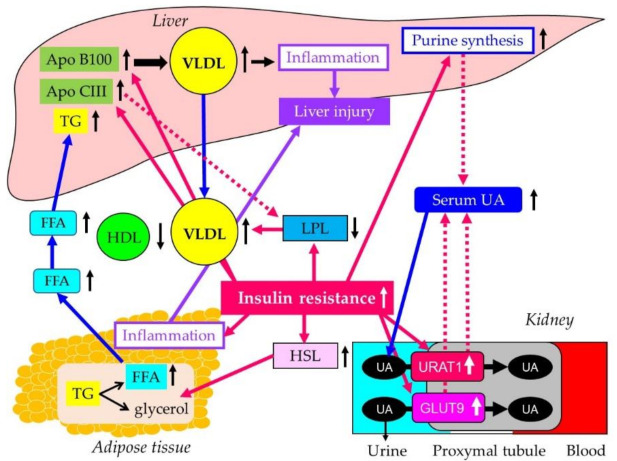
Possible molecular mechanisms for development of Atherogenic Dyslipidemia, liver dysfunction and hyperuricemia in insulin resistance. FFA, free fatty acid; GLUT9, glucose transporter 9; HDL, high-density lipoprotein; HSL, hormone sensitive lipase; LPL, lipoprotein lipase; TG, triglyceride; UA, uric acid; URAT1, urate transporter 1; VLDL, very-low density-lipoprotein.

Possible molecular mechanisms for development of atherogenic dyslipidemia, liver dysfunction and hyperuricemia in insulin resistance are shown in Figure 6 [22,23]. 

Insulin resistance increases activity and expression of hormone sensitive lipase (HSL) in adipose tissue, which catalyzes the breakdown of TG, releasing free fatty acid (FFA) [24]. Insulin promotes apo B100 degradation, and hepatic insulin resistance reduces apo B100 degradation [25]. Furthermore, insulin resistance is associated with elevated hepatic apo CIII production [26]. In the insulin resistant-state, increased FFA entry to liver and reduced degradation of apoB100, and elevated apo CIII levels may elevate hepatic production of TG-rich lipoprotein, very-low density-lipoprotein (VLDL). Insulin resistance also decreases the activity of LPL, the rate-limiting enzyme of the catabolism of TG-rich lipoproteins such as VLDL [27]. The formation of HDL is related to the catabolism of TG-rich lipoproteins by LPL [28]. Therefore, reduced LPL activity increases VLDL, and reduces HDL. Apo CIII inhibits LPL activity [29], which leads to further increase of VLDL and decrease of HDL. 

NAFLD is characterized by excess accumulation of TG in the hepatocyte due to both increased inflow of FFA and de novo hepatic lipogenesis [30]. Insulin resistance is the major mechanism in the development and progression of NAFLD. Overexpression of apo CIII produced NAFLD-like features, including increased liver lipid content, decreased antioxidant power, increased expression of inflammatory cytokines and increased cell death [31]. A high-fat diet further induced glucose intolerance, marked increases in inflammatory cytokines, cell death and apoptosis in apo CIII overexpressed mice. Fenofibrate treatment reversed several of the effects associated with diet and apo CIII expression but did not normalize inflammatory traits even when liver lipid content was fully corrected, suggesting that apo CIII and/or hypertriglyceridemia plays a major role in liver inflammation and cell death. Therefore, atherogenic dyslipidemia induced by insulin resistant may contribute to the development of NAFLD. Cytokines secreted by adipocytes, such as tumor necrosis factor-α, transforming growth factor-β, and interleukin-6, are implicated in the development of NAFLD [32].

Hyperuricemia is significantly associated with the development and severity of metabolic syndrome and insulin resistance [33]. The meta-analysis showed that higher serum UA levels led to an increased risk of metabolic syndrome regardless of the study characteristics, and were consistent with a linear dose-response relationship [34]. Magnitude of insulin resistance and serum UA concentration were significantly related, and insulin resistance was also inversely related to urinary UA clearance [35]. The influences of insulin resistance on UA metabolism are shown in Figure 6. Renal UA reabsorption is mainly mediated by urate transporter 1 (URAT1) and glucose transporter 9 (GLUT9) [33,36,37,38]. An increased protein level of URAT1 was observed in obesity/metabolic syndrome model mice [39]. Upon high-purine load, insulin resistance enhances UA reabsorption as manifested by up-regulated URAT1 expression and reduces UA excretion in the Otsuka-Long-Evans-Tokushima fatty rats [40]. 

In kidney tissue of Sprague-Dawley rats induced with metabolic syndrome, gene expression of GLUT9 was significantly upregulated [41]. Immunohistochemical study showed a significant increase of GLUT9 by more than three-fold. GLUT9 is a high-capacity urate transporter expressed in the proximal renal tubular cell, which reportedly also transports glucose and fructose [42]. Glycolytic disturbances were observed in insulin-resistant and hyper-uricemic states [43]. Diversion of glycolytic intermediates toward ribose-5-phosphate, phosphor-ribosyl-pyrophosphate, and UA will follow if there is diminished activity of glyceraldehyde-3-phosphate dehydrogenase (GA3PDH), which is regulated by insulin. Intrinsic defects in GA3PDH and a loss of its responsiveness to insulin can explain the association between insulin resistance and hyperuricemia by elevation of de novo purine synthesis [33]. Increased UA reabsorption by increased expression of URAT1 and GLUT9 and glycolytic disturbance may contribute to the development of hyperuricemia in insulin resistance.

An appropriate treatment of the components of metabolic syndrome is crucial to reduce hepatic morbidity and mortality. The most important class of medications to manage atherogenic dyslipidemia due to the metabolic syndrome can be fibrates because fibrates were associated with a greater reduction in TG, and a greater increase in HDL-C [7]. Pemafibrate is a novel member of the selective PPARα modulator family that was designed to have a higher PPARα agonistic activity and selectivity than existing agonists (such as fibrates) [5,14]. Our study demonstrated that pemafibrate significantly reduced serum TG and non-HDL-C and raised HDL-C. The activation of PPARα increases the catabolism of FA. In the liver, it activates their beta-oxidation [44]. The effects that PPARα exerts on TG-rich lipoproteins is due to their stimulation of LPL and repression of apolipoprotein CIII expression, while the effects on HDL depend upon the regulation of apolipoproteins AI and AII. In present study, a significant increase in LDL-C was observed in patients who did not receive SGLT2i; however, this was not observed in patients who received SGLT2i. Our previous study showed that SGLT2i decreased LDL-C [18], therefore the presence or absence of the combination with SGLT2i may be associated with this difference, which should be studied in the future. 

Serum ALT and GGT are indicators of liver damage [45]. Serum concentration of albumin reflects liver synthetic function [45]. Our study showed that pemafibrate improved ALT, GGT and albumin along with serum lipids, suggesting a significant beneficial effect of pemafibrate on liver damage and hepatic synthetic function. These improvements were observed in patients who did not receive SGLT2i, therefore these improvements may be due to pemafibrate. Interestingly, a significant decrease in AST was observed only in patients who received SGLT2i. Since SGLT2i improve liver function [17,18,19,20], synergism and/or the possible interaction between pemafibrate and SGLT2i may be associated with a decrease in AST, which still needs further studies. Accumulated data suggest that NAFLD is induced by abnormal hepatic lipid metabolism such as an increase of intra-hepatic fat content [46]. Fibrates reduce hepatic TG synthesis by decreasing apo C-III production, via PPARα [7]. Further, the treatment with PPARα agonist simulated the expression of enzymes involved in FA oxidation leading to a concomitant decrease of hepatic TG levels [47]. PPARα agonistic activity reduces serum TG by lowering hepatic TG production and by elevated FA oxidation [5,6,7,47], which may reduce intra-hepatic fat content and result in an improvement of liver function. A potent inducer of PPARα stimulated beta-oxidation in a model of steatohepatitis, and induced a complete clearance of steatosis together with a significant reduction of oxidative stress and oxidative injuries and prevention of inflammation and fibrosis [48]. Pemafibrate can be the promising therapeutic option for NAFLD.

**Figure 7:**
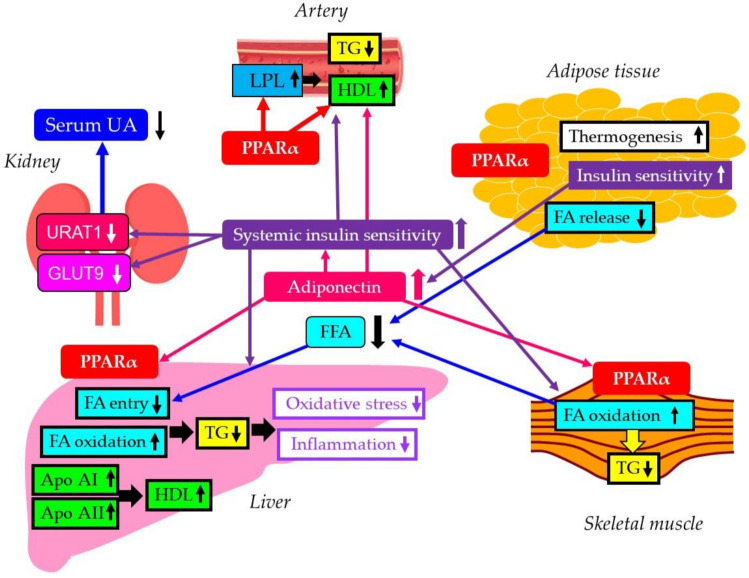
A possible improvement of the crosstalk between energy-demanding tissues induced by pemafibrate. FA, fatty acid; GLUT9, glucose transporter 9; HDL, high-density lipoprotein; LPL, lipoprotein lipase; PPARα, peroxisome proliferator-activated receptor alpha; TG, triglyceride; URAT1, urate transporter 1.

In addition to such direct effects of pemafibrate on liver, a possible improved crosstalk between energy-demanding tissues induced by pemafibarate may be also associated with an amelioration of liver function (Figure 7). 

Although PPARγ is a predominant PPAR in adipose tissue, the activation of PPARα is associated with a beneficial change in the quality of adipocytes. White adipose tissue (WAT) remodeling in obesity results in inflammation, insulin resistance, enhanced lipolysis, ectopic lipid accumulation and reduced energy expenditure [49]. WAT is currently considered as a metabolically active organ of an endocrine nature [50]. The secretion of adipokines and their roles in the regulation of energy homeostasis, inflammatory response, and glucose tolerance are altered in obese individuals [51]. Brown fat expansion and/or activation results in increased energy expenditure and a negative energy balance in mice and limits weight gain [52]. Brown fat is also able to utilize blood glucose and lipid and results in improved glucose metabolism and serum lipids independent of weight loss [52]. PPARα agonists can induce browning of white adipose tissue. Browning, verified by uncoupling protein 1 (UCP1) positive beige cells and enhanced body temperature, was only observed in PPARα treated obese mice [53]. Cytokines secreted by adipocytes, such as tumor necrosis factor-α, transforming growth factor-β, and interleukin-6, are implicated in the development of NAFLD [32]. The WAT after browning produces fewer cytokines, which may improve liver function. Further, PPARα agonist enhances adiponectin production [54], which may be also beneficially associated with liver function [55]. 

The activation of PPAR-α markedly stimulated the muscle expression of two key enzymes involved in lipid oxidation, namely carnitine-palmitoyl transferase and acyl-CoA oxidase [56]. Moreover, the liver and muscle tissue TG content were significantly reduced after the PPAR-α treatment [56]. The euglycemic-hyper-insulinemic clamp demonstrated a marked improvement in the hepatic insulin sensitivity and an increase in the whole body insulin sensitivity [56]. Therefore, elevated FA oxidation by pemafibrate and resulting reduced serum FA and FA entry to liver may be associated with an improvement of liver function. An increased systemic insulin sensitivity may be also beneficially associated with liver function. A reduction of HbA1c after the start of pemafibrate in the analysis of all patients in our study may reflect an improvement of systemic insulin sensitivity by pemafibrate. Especially, significant decreases in plasma glucose and HbA1c were observed in patients who received SGLT2i. The synergism and/or the possible interaction between pemafibrate and SGLT2i may be associated with an improvement of glucose metabolism, which still warrant further studies.

Increased UA reabsorption by increased expression of URAT1 and GLUT9 and glycolytic disturbance may contribute to the development of hyperuricemia in insulin resistance. Our study showed that serum UA significantly decreased at 12 months after the start of pemafibrate as compared with baseline. A significant decrease in serum UA was observed in patients who did not receive SGLT2i, support that pemafibrate. One of fibrates, fenofibrate, decreased serum UA levels by increasing its urinary excretion, most likely through the inhibition of URAT1 by fenofibric acid, its major metabolite [57]. The meta-analyses showed a significant reduction in plasma UA concentrations following fenofibrate therapy [58,59]. However, reduction of serum UA by pemafibrate was not reported. Fenofibrate has URAT1 inhibitory effect, but pemafibrate may not have such effect, which was supported by our recent study that showed a significant increase of serum UA after the switching from fenofibrate to pemafibrate [60]. In this study, pemafibrate may decrease serum UA by reducing over-expressed URAT1 and GLUT9 due to insulin resistance, and by improving systemic insulin resistance, which may be supported by a significant and positive correlation between changes in serum UA and changes in TG at 12 months after the start of pemafibrate. 

Limitations of the study need to be addressed. This is a cross-sectional study, limiting inferences of causality and its direction. Although we did not intentionally change treatments for diabetes and hypertension, hyperuricemia and other lipid-lowering drugs during the study period, we cannot deny the beneficial role of the concomitant assumption of other drugs including aspects of synergism and/or the possible interaction between pemafibrate and other treatments against the different components of metabolic syndrome such as SGLT2i in the present data. To elucidate this, further studies, preferentially a prospective randomized placebo control study including a large number of patients, should be performed in the future. 

## 5. Conclusions

We observed a significant improvement in liver function, HbA1c and serum UA along with an amelioration of dyslipidemia after the start of pemafibrate. 

## Figures and Tables

**Table 1:** Baseline characteristics for patients who had taken pemafibrate (n = 246).

Clinical Characteristics	
Age (years old)	60.0 ± 15.9
Gender (male/female)	140/106
Body weight (mean ± SD, kg)	73.7 ± 16.6
Body mass index (mean ± SD, kg/m^2^)	27.0 ± 5.5
Systolic blood pressure (mean ± SD, mmHg)	134.1 ± 17.3
Diastolic blood pressure (mean ± SD, mmHg)	78.4 ± 13.9
**Comorbidities**	
Type 2 diabetes (n, %)	121, 49.2%
Hypertension (n, %)	113, 46.0%
Hyperuricemia (n, %)	73, 29.7%
**Treatments for type 2 diabetes**	
Dipeptidyl peptidase-4 inhibitors (n, %)	67, 27.2%
Metformin (n, %)	76, 30.9%
Sodium-glucose co-transporter 2 inhibitors (n, %)	63, 25.6%
Pioglitazone (n, %)	18, 7.3%
Insulin (n, %)	23, 9.3%
Glucagon-like peptide 1 receptor-agonists (n, %)	17, 6.9%
**Treatments for hypertension**	
Angiotensin receptor blockers (n, %)	88, 35.8%
Calcium antagonists (n, %)	95, 38.6%
Diuretics (n, %)	14, 5.7%
α, β-blockers (n, %)	31, 12.6%
**Treatments for dyslipidemia**	
Statins (n, %)	85, 34.6%
Ezetimibe (n)	43, 17.5%
Eicosapentaenoic acid (n, %)	30, 12.2%
Fenofibrate (n, %)	20, 8.1%
**Treatments for hyperuricemia**	
Febuxostat (n, %)	22, 8.9%
Topiroxostat (n, %)	8, 3.3%
Allopurinol (n, %)	12, 4.9%

**Table 2:** Changes in metabolic parameters after the strart of pemafibrate in all patients (n = 246).

	Baseline	After 3 Months	After 6 Months	After 12 Months
Body weight (kg)	73.7 ± 16.7	72.9 ± 15.2	73.1 ± 14.0	73.3 ± 14.4
Systolic blood pressure (mmHg)	134.1 ± 17.3	133.0 ± 14.5	133.9 ± 14.2	132.0 ± 14.2
Diastolic blood pressure (mmHg)	78.4 ± 13.9	78.6 ± 12.0	78.7 ± 12.0	77.6 ± 12.0
TG (mg/dL)	411.0 ± 349.5	231.1 ± 221.9 *	214.2 ± 171.6 *	206.1 ± 147.8 *
HDL-C (mg/dL)	46.0 ± 13.7	51.0 ± 13.3 *	50.5 ± 12.7 *	51.4 ± 13.2 *
LDL-C (mg/dL)	105.6 ± 35.3	110.7 ± 33.1	107.0 ± 31.6	109.1 ± 32.6
Non-HDL-C (mg/dL)	170.6 ± 56.3	147.3 ± 39.5 *	141.2 ± 36.8 *	138.6 ± 35.5 *
AST (IU/L)	36.5 ± 58.1	33.9 ± 48.4 *	29.7 ± 20.2 *	28.9 ± 21.2
ALT (IU/L)	40.6 ± 42.3	33.5 ± 32.4 *	30.0 ± 24.5 *	30.5 ± 31.3 *
GGT (IU/L)	80.4 ± 129.3	50.5 ± 70.7 *	57.8 ± 87.7 *	53.0 ± 74.6 *
Albumin (g/dL)	4.25 ± 0.44	4.43 ± 0.36 *	4.42 ± 0.37 *	4.42 ± 0.38 *
UA (mg/dL)	6.0 ± 1.6	6.0 ± 1.5	5.8 ± 1.5	5.7 ± 1.2 *
eGFR (mL/min/1.73m^2^)	72.6 ± 27.7	72.1 ± 28.6	71.5 ± 23.7	68.1 ± 21.9
Plasma glucose (mg/dL)	142.4 ± 49.5	137.6 ± 43.8	138.3 ± 48.4	135.0 ± 38.2
HbA1c (%)	6.8 ± 1.3	6.6 ± 1.0 *	6.6 ± 0.9	6.6 ± 0.9

Values show mean ± SD. * *p* < 0.05 vs. baseline. ALT, alanine aminotransferase; AST, aspartate aminotransferase; eGFR, estimated glomerular filtration rate; GGT, gamma-glutamyl transferase; HDL-C, high-density lipoprotein-cholesterol; LDL-C, low-density lipoprotein-cholesterol; Non-HDL-C, non-high-density lipoprotein-cholesterol; TG, triglyceride, UA, uric acid.

**Table 3:** Changes in metabolic parameters after the strart of pemafibrate in patients with and without the treatment using SGLT2i.

	Patients without the Treatment Using SGLT2i (n = 183)				Patients with the Treatment Using SGLT2i (n = 63)			
	Baseline	After 3 Months	After 6 Months	After 12 Months	Baseline	After 3 Months	After 6 Months	After 12 Months
Body weight (kg)	69.4 ± 17.6	71.6 ± 15.8	72.3 ± 14.9	73.2 ± 15.4	77.3 ± 14.2	76.2 ± 13.3	75.0 ± 12.1	73.1 ± 11.6
Systolic blood pressure (mmHg)	133.4 ± 18.3	132.2 ± 14.0	133.5 ± 14.2	132.7 ± 14.9	133.1 ± 17.9	133.5 ± 15.8	133.8 ± 14.2	130.7 ± 11.5
Diastolic blood pressure (mmHg)	77.4 ± 12.9	77.4 ± 12.4	77.4 ± 11.8	77.2 ± 12.7	81.2 ± 14.4	81.0 ± 10.9	80.9 ± 12.1	79.4 ± 10.5
TG (mg/dL)	376.6 ± 342.3	208.0 ± 167.1 *	202.3 ± 139.6 *	196.3 ± 143.1 *	422.5 ± 327.4	298.0 ± 323.7 *	259.7 ± 248.2 *	235.4 ± 158.8 *
HDL-C (mg/dL)	47.0 ± 15.1	51.9 ± 13.5 *	51.1 ± 13.5 *	51.8 ± 13.2 *	46.7 ± 12.2	49.3 ± 13.0 *	49.4 ± 11.3 *	51.7 ± 11.3 *
LDL-C (mg/dL)	107.1 ± 33.5	114.0 ± 34.8 *	111.4 ± 34.8	109.3 ± 34.2 *	105.7 ± 35.3	104.3 ± 29.3	99.0 ± 28.9	104.4 ± 29.5
Non-HDL-C (mg/dL)	166.5 ± 50.5	148.1 ± 39.6 *	143.4 ± 37.9 *	138.1 ± 38.1 *	167.4 ± 60.6	143.0 ± 39.1 *	130.5 ± 31.0 *	133.2 ± 30.1 *
AST (IU/L)	35.9 ± 60.9	35.9 ± 53.9	31.3 ± 23.0	31.3 ± 29.8	35.0 ± 26.6	28.8 ± 25.2 *	26.9 ± 13.4	27.8 ± 15.3 *
ALT (IU/L)	38.1 ± 41.5	33.4 ± 28.3 *	30.7 ± 25.8 *	30.3 ± 32.1 *	41.4 ± 34.5	34.2 ± 41.9 *	29.1 ± 19.8 *	32.4 ± 30.1 *
GGT (IU/L)	80.3 ± 143.6	52.8 ± 78.3 *	61.4 ± 106.3 *	55.7 ± 135.2 *	88.3 ± 118.8	43.6 ± 38.9 *	55.9 ± 66.9 *	78.4 ± 110.9 *
Albumin (g/dL)	4.22 ± 0.42	4.43 ± 0.36 *	4.41 ± 0.37 *	4.44 ± 0.33 *	4.33 ± 0.45	4.43 ± 0.36 *	4.47 ± 0.37	4.40 ± 0.47
UA (mg/dL)	6.0 ± 1.7	6.1 ± 1.5	5.9 ± 1.6	5.8 ± 1.3 *	5.8 ± 1.5	5.9 ± 1.6	5.5 ± 1.2	5.5 ± 1.0
eGFR (mL/min/1.73m^2^)	69.8 ± 24.7	69.5 ± 18.6	70.1 ± 19.3	66.2 ± 18.6	78.5 ± 34.3	81.9 ± 44.1	79.2 ± 33.7	75.7 ± 29.5
Plasma glucose (mg/dL)	134.7 ± 51.3	130.1 ± 42.3	132.4 ± 47.9	130.4 ± 38.7	163.3 ± 49.7	153.2 ± 42.9 *	151.7 ± 46.1	146.2 ± 34.2
HbA1c (%)	6.3 ± 1.1	6.3 ± 0.8	6.3 ± 0.8	6.3 ± 0.8	7.6 ± 1.3	7.2 ± 1.0 *	7.2 ± 0.9	7.2 ± 0.9

Values show mean ± SD. * *p* < 0.05 vs. baseline. ALT, alanine aminotransferase; AST, aspartate aminotransferase; eGFR, estimated glomerular filtration rate; GGT, gamma-glutamyl transferase; HDL-C, high-density lipoprotein-cholesterol; LDL-C, low-density lipoprotein-cholesterol; Non-HDL-C, non-high-density lipoprotein-cholesterol; SGLT2i, sodium-glucose co-transporter 2 inhibitors; TG, triglyceride, UA, uric acid.

## Data Availability

The data supporting the findings of this study are available from the corresponding author upon reasonable request.

## References

[1] Alberti K.G., Eckel R.H., Grundy S.M., Zimmet P.Z., Cleeman J.I., Donato K.A., Fruchart J.C., James W.P., Loria C.M., Smith S.C. (2009). Harmonizing the metabolic syndrome: A joint interim statement of the international diabetes federation task force on epidemiology and prevention; National Heart, Lung, and Blood Institute; American Heart Association; World Heart Federation; International Atherosclerosis Society; and International Association for the Study of Obesity. Circulation.

[2] Motojima K. (1993). Peroxisome Proliferator-Activated Receptor (PPAR): Structure, Mechanisms of Activation and Diverse Functions. Cell Struct. Funct..

[3] Wójtowicz S., Strosznajder J.B., Jeżyna M. (2020). The Novel Role of PPAR Alpha in the Brain: Promising Target in Therapy of Alzheimer’s Disease and Other Neurodegenerative Disorders. Neurochem. Res..

[4] Issemann I., Green S. (1990). Activation of a member of the steroid hormone receptor superfamily by peroxisome proliferators. Nature.

[5] Fruchart J.C. (2013). Selective peroxisome proliferator-activated receptor α modulators (SPPARMα): The next generation of peroxisome proliferator-activated receptor α-agonists. Cardiovasc. Diabetol..

[6] Braissant O., Foufelle F., Scotto C., Daua M., Wahli W. (1996). Differential expression of peroxisome proliferator-activated receptors (PPARs): Tissue distribution of PPAR-alpha, -beta, and -gamma in the adult rat. Endocrinology.

[7] Staels B., Dallongeville J., Auwerx J., Schoonjans K., Leitersdorf E., Fruchart J.-C. (1998). Mechanism of action of fibrates on lipid and lipoprotein metabolism. Circulation.

[8] Loomba R.S., Arora R. (2010). Prevention of Cardiovascular Disease Utilizing Fibrates-A Pooled Meta-analysis. Am. J. Ther..

[9] Rosenson R.S. (2008). Fenofibrate: Treatment of hyperlipidemia and beyond. Expert Rev. Cardiovasc. Ther..

[10] Feingold K., Grunfeld C., Groot L.J., Chrousos G., Dungan K., Feingold K.R., Grossman A., Hershman J.M., Koch C., Korbonits M. (2017). Triglyceride Lowering Drugs. Endotext.

[11] Wang D., Liu B., Tao W., Hao Z., Liu M. (2015). Fibrates for secondary prevention of cardiovascular disease and stroke. Cochrane Database Syst. Rev..

[12] Ahmad J., Odin J.A., Hayashi P.H., Chalasani N., Fontana R.J., Barnhart H., Cirulli E.T., Kleiner D.E., Hoofnagle J.H. (2017). Identification and Characterization of Fenofibrate-Induced Liver Injury. Am. J. Dig. Dis..

[13] Davidson M.H., Armani A., McKenney J.M., Jacobson T. (2007). Safety Considerations with Fibrate Therapy. Am. J. Cardiol..

[14] Yamazaki Y., Abe K., Toma T., Nishikawa M., Ozawa H., Okuda A., Araki T., Oda S., Inoue K., Shibuya K. (2007). Design and synthesis of highly potent and selective human peroxisome proliferator-activated receptor α agonists. Bioorg. Med. Chem. Lett..

[15] Yokote K., Yamashita S., Arai H., Araki E., Suganami H., Ishibashi S., on behalf of the KSGOB (2019). Long-term efficacy and safety of pemafibrate, a novel selective peroxisome proliferator-activated receptor-alpha modulator (SPPARMalpha), in dyslipidemic patients with renal impairment. Int. J. Mol. Sci..

[16] Yamashita S., Masuda D., Matsuzawa Y. (2019). Clinical applications of a novel selective PPARalpha modulator, pemafibrate, in dyslipidemia and metabolic diseases. J. Atheroscler. Thromb..

[17] Yanai H., Hakoshima M., Adachi H., Katsuyama H. (2021). Multi-Organ Protective Effects of Sodium Glucose Cotransporter 2 Inhibitors. Int. J. Mol. Sci..

[18] Katsuyama H., Hamasaki H., Adachi H., Moriyama S., Kawaguchi A., Sako A., Mishima S., Yanai H. (2016). Effects of Sodium-Glucose Cotransporter 2 Inhibitors on Metabolic Parameters in Patients with Type 2 Diabetes: A Chart-Based Analysis. J. Clin. Med. Res..

[19] Yanai H., Hakoshima M., Adachi H., Kawaguchi A., Waragai Y., Harigae T., Masui Y., Kakuta K., Hamasaki H., Katsuyama H. (2017). Effects of Six Kinds of Sodium-Glucose Cotransporter 2 Inhibitors on Metabolic Parameters, and Summarized Effect and Its Correlations with Baseline Data. J. Clin. Med. Res..

[20] Katsuyama H., Hakoshima M., Iijima T., Adachi H., Yanai H. (2020). Effects of Sodium-Glucose Cotransporter 2 Inhibitors on Hepatic Fibrosis in Patients with Type 2 Diabetes: A Chart-Based Analysis. J. Endocrinol. Metab..

[21] Fan J.-G., Peng Y.-D. (2007). Metabolic syndrome and non-alcoholic fatty liver disease: Asian definitions and Asian studies. Hepatobiliary Pancreat. Dis. Int..

[22] Yanai H., Hirowatari Y., Yoshida H. (2019). Diabetic dyslipidemia: Evaluation and mechanism. Glob. Health Med..

[23] Yanai H., Hirowatari Y., Ito K., Kurosawa H., Tada N., Yoshida H. (2016). Understanding of Diabetic Dyslipidemia by Using the Anion-Exchange High Performance Liquid Chromatography Data. J. Clin. Med. Res..

[24] Sztalryd C., Kraemer F. (1995). Regulation of hormone-sensitive lipase in streptozotocin-induced diabetic rats. Metabolism.

[25] Taghibiglou C., Carpentier A., Van Iderstine S.C., Chen B., Rudy D., Aiton A., Lewis G.F., Adeli K. (2001). Mechanisms of hepatic very low density lipoprotein overproduction in insulin resistance. Evidence for enhanced lipoprotein assembly, reduced intracellular apoB degradation, and increased microsomal triglyceride transfer protein in a fructose-fed hamster model. J. Biol. Chem..

[26] Chen M., Breslow J.L., Li W., Leff T. (1994). Transcriptional regulation of the apoC-III gene by insulin in diabetic mice: Correlation with changes in plasma triglyceride levels. J. Lipid Res..

[27] Fisher E.A. (2012). The degradation of apolipoprotein B100: Multiple opportunities to regulate VLDL triglyceride production by different proteolytic pathways. Biochim. Biophys. Acta (BBA) Mol. Cell Biol. Lipids.

[28] Nikkilä E.A., Taskinen M.-R., Kekki M. (1978). Relation of plasma high-density lipoprotein cholesterol to lipoprotein-lipase activity in adipose tissue and skeletal muscle of man. Atherosclerosis.

[29] Ooi E.M.M., Barrett P.H.R., Chan D.C., Watts G.F. (2008). Apolipoprotein C-III: Understanding an emerging cardiovascular risk factor. Clin. Sci..

[30] Engin A. (2017). Non-Alcoholic Fatty Liver Disease. Adv. Exp. Med. Biol..

[31] Paiva A.A., Raposo H.F., Wanschel A.C., Nardelli T.R., Oliveira H.C. (2017). Apolipoprotein CIII Overexpression-Induced Hypertriglyceridemia Increases Nonalcoholic Fatty Liver Disease in Association with Inflammation and Cell Death. Oxid. Med. Cell. Longev..

[32] Giby V.G., Ajith T.A. (2014). Role of adipokines and peroxisome proliferator-activated receptors in nonalcoholic fatty liver disease. World J. Hepatol..

[33] Yanai H., Adachi H., Hakoshima M., Katsuyama H. (2021). Molecular Biological and Clinical Understanding of the Pathophysiology and Treatments of Hyperuricemia and Its Association with Metabolic Syndrome, Cardiovascular Diseases and Chronic Kidney Disease. Int. J. Mol. Sci..

[34] Yuan H., Yu C., Li X., Sun L., Zhu X., Zhao C., Zhang Z., Yang Z. (2015). Serum Uric Acid Levels and Risk of Metabolic Syndrome: A Dose-Response Meta-Analysis of Prospective Studies. J. Clin. Endocrinol. Metab..

[35] Facchini F., Chen Y.D., Hollenbeck C.B., Reaven G.M. (1991). Relationship between resistance to insulin-mediated glucose uptake, urinary uric acid clearance, and plasma uric acid concentration. JAMA.

[36] Enomoto A., Kimura H., Chairoungdua A., Shigeta Y., Jutabha P., Cha S.H., Hosoyamada M., Takeda M., Sekine T., Igarashi T. (2002). Molecular identification of a renal urate–anion exchanger that regulates blood urate levels. Nature.

[37] Li S., Sanna S., Maschio A., Busonero F., Usala G., Mulas A., Lai S., Dei M., Orrù M., Albai G. (2007). The GLUT9 gene is associated with serum uric acid levels in Sardinia and Chianti cohorts. PLoS Genet..

[38] Vitart V., Rudan I., Hayward C., Gray N., Floyd J., Palmer C., Knott S.A., Kolcic I., Polasek O., Graessler J. (2008). SLC2A9 is a newly identified urate transporter influencing serum urate concentration, urate excretion and gout. Nat. Genet..

[39] Doshi M., Takiue Y., Saito H., Hosoyamada M. (2011). The Increased Protein Level of URAT1 was Observed in Obesity/Metabolic Syndrome Model Mice. Nucleosides Nucleotides Nucleic Acids.

[40] Miao Z., Yan S., Wang J., Wang B., Li Y., Xing X., Yuan Y., Meng D., Wang L., Gu J. (2009). Insulin resistance acts as an independent risk factor exacerbating high-purine diet induced renal injury and knee joint gouty lesions. Inflamm. Res..

[41] Ng H.-Y., Lee Y.-T., Kuo W.-H., Huang P.-C., Lee W.-C., Lee C.-T. (2018). Alterations of Renal Epithelial Glucose and Uric Acid Transporters in Fructose Induced Metabolic Syndrome. Kidney Blood Press. Res..

[42] Caulfield M.J., Munroe P.B., O’Neill D., Witkowska K., Charchar F.J., Doblado M., Evans S., Eyheramendy S., Onipinla A., Howard P. (2008). SLC2A9 is a high-capacity urate transporter in humans. PLoS Med..

[43] Leyva F., Wingrove C.S., Godsland I.F., Stevenson J.C. (1998). The glycolytic pathway to coronary heart disease: A hypothesis. Metabolism.

[44] Neve B., Fruchart J.-C., Staels B. (2000). Role of the peroxisome proliferator-activated receptors (PPAR) in atherosclerosis. Biochem. Pharmacol..

[45] Ge P.-L., Du S.-D., Mao Y.-L. (2014). Advances in preoperative assessment of liver function. Hepatobiliary Pancreat. Dis. Int..

[46] Guo R., Liong E.C., So K.F., Fung M.-L., Tipoe G.L. (2015). Beneficial mechanisms of aerobic exercise on hepatic lipid metabolism in non-alcoholic fatty liver disease. Hepatobiliary Pancreat. Dis. Int..

[47] Kim H., Haluzik M., Asghar Z., Yau D., Joseph J.W., Fernandez A.M., Reitman M.L., Yakar S., Stannard B., Heron-Milhavet L. (2003). Peroxisome Proliferator–Activated Receptor-α Agonist Treatment in a Transgenic Model of Type 2 Diabetes Reverses the Lipotoxic State and Improves Glucose Homeostasis. Diabetes.

[48] Leclercq I.A. (2007). Pathogenesis of steatohepatitis: Insights from the study of animal models. Acta Gastro Enterol. Belg..

[49] Ahima R.S. (2006). Adipose Tissue as an Endocrine Organ. Obesity.

[50] Giralt M., Villarroya F. (2013). White, Brown, Beige/Brite: Different Adipose Cells for Different Functions?. Endocrinology.

[51] Fonseca-Alaniz M.H., Takada J., Alonso-Vale M.I., Lima F.B. (2007). Adipose tissue as an endocrine organ: From theory to practice. J. Pediatr..

[52] Kim S.H., Jorge Plutzky J. (2016). Brown Fat and Browning for the Treatment of Obesity and Related Metabolic Disorders. Diabetes Metab. J..

[53] Rachid T.L., Silva-Veiga F.M., Graus-Nunes F., Bringhenti I., Mandarim-de-Lacerda C.A., Souza-Mello V. (2018). Differential actions of PPAR-alpha and PPAR-beta/delta on beige adipocyte formation: A study in the subcutaneous white adipose tissue of obese male mice. PLoS ONE.

[54] Maia-Fernandes T., Roncon-Albuquerque R., Leite-Moreira A.F. (2008). Cardiovascular actions of adiponectin: Pathophysiologic implications. Rev. Port. Cardiol..

[55] Yanai H., Yoshida H. (2019). Beneficial Effects of Adiponectin on Glucose and Lipid Metabolism and Atherosclerotic Progression: Mechanisms and Perspectives. Int. J. Mol. Sci..

[56] Haluzík M.M., Haluzík M. (2005). PPAR-alpha and insulin sensitivity. Physiol. Res..

[57] Uetake D., Ohno I., Ichida K., Yamaguchi Y., Saikawa H., Endou H., Hosoya T. (2010). Effect of fenofibrate on uric acid metabolism and urate transporter 1. Intern. Med..

[58] Zhang J., Ji X., Dong Z., Lu J., Zhao Y., Li R., Li C., Chen Y. (2021). Impact of fenofibrate therapy on serum uric acid concentrations: A review and meta-analysis. Endocr. J..

[59] Derosa G., Maffioli P., Sahebkar A. (2015). Plasma uric acid concentrations are reduced by fenofibrate: A systematic review and meta-analysis of randomized placebo-controlled trials. Pharmacol. Res..

[60] Yanai H., Katsuyama H., Hakoshima M. (2021). A Significant Increase of Estimated Glomerular Filtration Rate After Switching From Fenofibrate to Pemafibrate in Type 2 Diabetic Patients. Cardiol. Res..

